# Fuzzy Logic Based Edge Detection in Smooth and Noisy Clinical Images

**DOI:** 10.1371/journal.pone.0138712

**Published:** 2015-09-25

**Authors:** Izhar Haq, Shahzad Anwar, Kamran Shah, Muhammad Tahir Khan, Shaukat Ali Shah

**Affiliations:** 1 Institute of Mechatronics Engineering, University of Engineering and Technology, Peshawar, Pakistan; 2 Department of Mechanical Engineering, University of Engineering and Technology, Peshawar, Pakistan; Glasgow University, UNITED KINGDOM

## Abstract

Edge detection has beneficial applications in the fields such as machine vision, pattern recognition and biomedical imaging etc. Edge detection highlights high frequency components in the image. Edge detection is a challenging task. It becomes more arduous when it comes to noisy images. This study focuses on fuzzy logic based edge detection in smooth and noisy clinical images. The proposed method (in noisy images) employs a *3*×*3* mask guided by fuzzy rule set. Moreover, in case of smooth clinical images, an extra mask of contrast adjustment is integrated with edge detection mask to intensify the smooth images. The developed method was tested on noise-free, smooth and noisy images. The results were compared with other established edge detection techniques like Sobel, Prewitt, Laplacian of Gaussian (LOG), Roberts and Canny. When the developed edge detection technique was applied to a smooth clinical image of size *270*×*290* pixels having *24* dB ‘salt and pepper’ noise, it detected very few (*22)* false edge pixels, compared to Sobel (*1931*), Prewitt (*2741*), LOG (*3102*), Roberts (*1451*) and Canny (*1045*) false edge pixels. Therefore it is evident that the developed method offers improved solution to the edge detection problem in smooth and noisy clinical images.

## Introduction

Edges in an image are contours generated as a result of sudden or abrupt change in any of the (multiple) characteristics at pixel level. These changes could be observed due to alteration in colour, texture, shade or light absorption. These characteristics could further lead in estimating the orientation, size, depth and surface features in an image [1]. Edge detection has numerous applications in the field of robotics [2], medical image analysis [3], geographical science [4], pattern recognition [5], and military technology [6] etc. In medical images the role of edge detection is significant and has extensively been employed for the detection of structures and anomalies in computerized tomography (CT) scans, positron emission tomography (PET) scans and magnetic resonance images (MRI) [7]. It is often the case that these images embody high frequency noise or irrelevant data which inhibits the detection of continuous edge points [8], since edge itself is a composition of high frequency data. The noise generates false flags as they often mislead the algorithms for an edge.

Many techniques have been employed for the development of an optimum edge detection algorithm [9–14]. Each effort is guided by the motivation to overcome the limitations in previous methodologies. The conventional techniques incorporate the use of linear time invariant filters. These filters recognize an edge as an abrupt change of grey scale pixel intensities. The techniques are well established and computationally efficient. Canny [9], Sobel [10], Robert [11], Kirsch [12], Prewitt [13] and LOG [14], are based on the concept of spatial differential filters utilizing local gradient. These filters process the data in a relatively short time and are computationally optimized, however, they are susceptible to noise.

Jiange and Bunke [15] proposed an approximation of scan lines method for edge detection. The results achieved were considerably accurate and substantial in comparison to other segmentation techniques. A *5*×*5* kernel was developed by Genming and Bouzong [16] for the detection of edges in an image based on a fixed threshold level. However, their limitation was their inadaptability to regions with varying greyscale due to a fixed threshold point. Recent techniques incorporates methods developed for artificial neural networks [17], ant colony optimization [18], and genetic algorithms with particle swarm optimization [19].

Fuzzy Set theory is another technique that has been employed for edge detection [20–21].The method performs mathematical and logical reasoning based on approximations rather than crisp values. Therefore the technique significantly reduces the complexity of problems where fixed values cannot be attained or predicted. Kim et al. [22] proposed a methodology employing the use of a *3*×*3* kernel and a look up table. However, the technique could not adapt to challenging tests as it required manual tuning and configuration for each test. Sixteen fuzzy rules were defined for edge detection in a study conducted by Kaur et al. [23]. The results for edge detection were appreciable in images (with no noise) but performed poorly when noise was introduced. Further studies have been conducted in higher form of fuzzy logic especially fuzzy type-2 to accommodate greater uncertainties [24–26]. A theoretical perception suggests that higher order fuzzy rules set would compensate other limitations and effectively represent uncertainties. Unfortunately, the complexity of representation of model in fuzzy type-2 increases multi-folds.

To address these concerns this study is to develop a methodology that is able to detect edges effectively in smooth and noisy clinical images. Our technique employs a *3×3* mask guided by fuzzy rule set for edge detection in noisy images. Moreover, for smooth clinical images an extra mask of contrast adjustment is integrated with the edge detection mask based on fuzzy logic to intensify the smooth images. A robust filter was achieved as a result which is convenient to apply (invariant to noise and achieves optimal results).

The remaining article is organized in the following sections. Section 2 presents the developed methodology for edge detection followed by simulation results and discussion in section 3. Finally conclusions are drawn in section 4.

## Proposed Methodology

The Lady Reading Hospital (LRH) Peshawar, Pakistan medical staff explain the Magnetic Resonance Imaging (MRI) procedure to the patient. Subsequently, verbal consent was acquired (from the patient) prior to the MRI, and this was documented and added to the patient record. The data employed in this study provided by the LRH was completely anonymous and unidentified. Since the data is unidentified therefore the ethics committee of the LRH approved the study protocol and the method of consent.

**Fig 1:**
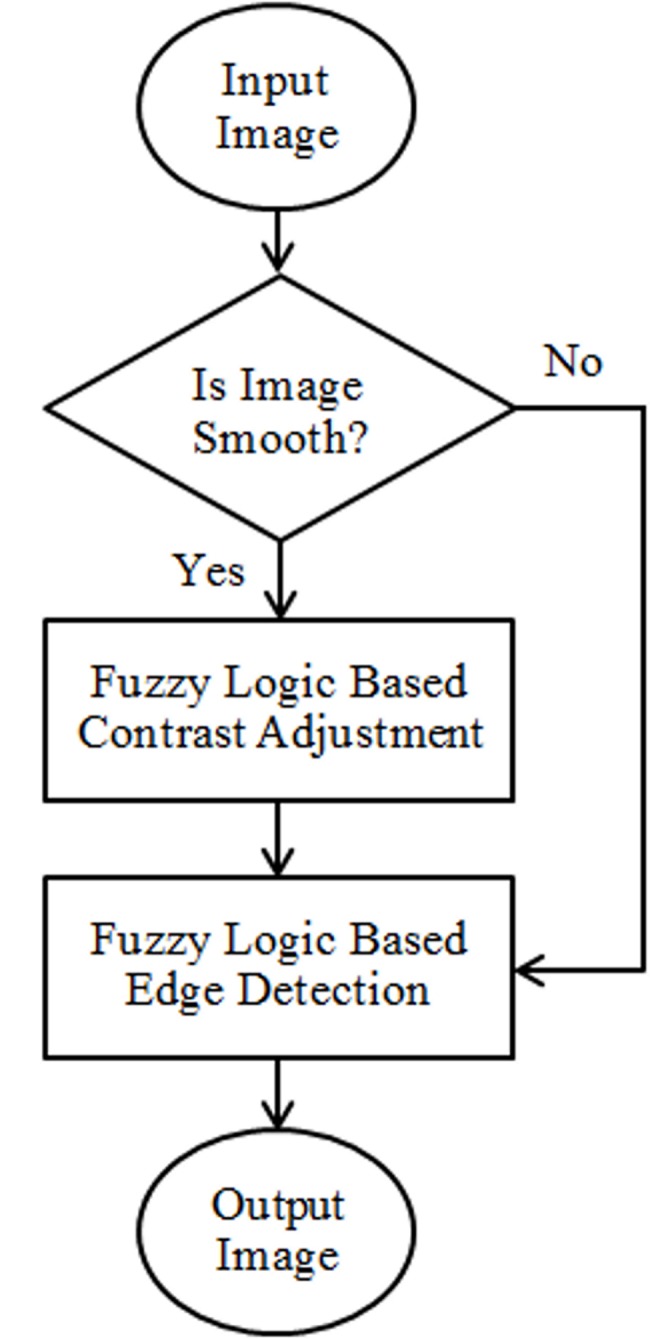
Work flow of the proposed edge detection technique.

The proposed edge detection algorithm for noisy and clinical images is based on a fuzzy inference system. A two mask technique was used to detect edges in greyscale images. For detection of edges in noisy images only one mask (edge detection mask) was used. However, for smooth clinical images an extra mask of contrast adjustment was integrated with the edge detection mask to intensify the image based on fuzzy logic. The workflow of the proposed methodology is shown in Fig 1.

### Edge Detection

**Fig 2:**
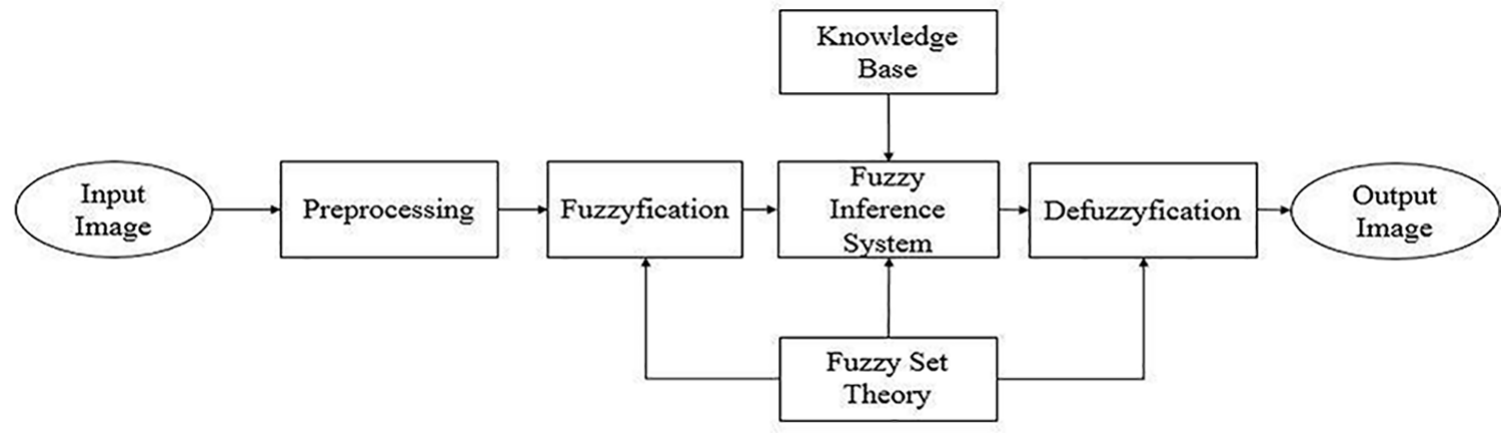
Block diagram of the developed edge detection approach through fuzzy logic.

The developed edge detection technique for noisy images is based on fuzzy logic. A *3*x*3* window mask was designed to take the greyscale values of neighborhood pixels from the input image. The greyscale values of the neighborhood pixels obtained from the mask were pre-processed prior to the fuzzy inference system. A fuzzy inference system was designed to take the processed values as an input. These values were subsequently converted into the fuzzy plane. A fuzzy rule base was defined to determine and show the edge pixels’ in the output image. The output of the system was calculated by the centroid method and defuzzification was performed based on Mamdani inference. The block diagram of the proposed fuzzy edge detection is shown in Fig 2.

### Window Mask

**Fig 3:**
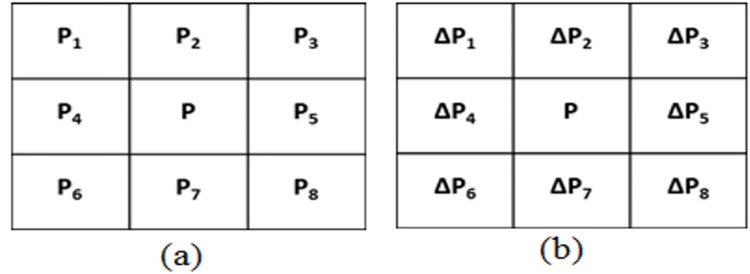
(a) Window mask, (b) Processed window mask.

A 3x3 window mask was designed for scanning the image, in the proposed approach as shown in Fig 3(A). The mask took the greyscale values, *P*_*j*_ of eight neighborhood pixels with the central pixel, *P* as the out pixel. The greyscale values obtained from the mask were pre-processed. Fig 3(B) shows the processed mask, where Δ*P*_*j*_ = |*P*_*j*_ − *P*| for j = 1, 2, 3… 8.

### Fuzzy membership functions

In fuzzy inference system, membership functions (MFs) play a key role. In the fuzzy set, fuzziness is measured using MFs as they are the key constituents of the fuzzy set theory. The type and shape of the MF should carefully be selected as they have effects on the fuzzy inference system. Trapezoidal MFs were used for the input data, because they exhibit reasonably improved results in comparison to other MFs [27–28]. Whereas, Gaussian MFs were used for the output data, because they are smooth and non-zero at all points. The standard trapezoidal membership function *T*_*rz*_ *F* [29] is expressed as:

$$T_{rz}F\;\left(w;r,s,t,u\right)=\;\{\begin{array}{l} 0\;\left(w<r\right)or\left(w>u\right)\\ \frac{z-r}{s-r}\;r\leq w\leq s\\ 1\;s\leq w\leq t\\ \frac{u-z}{u-t}\;t\leq w\leq u \end{array}$$ (1)

**Fig 4:**
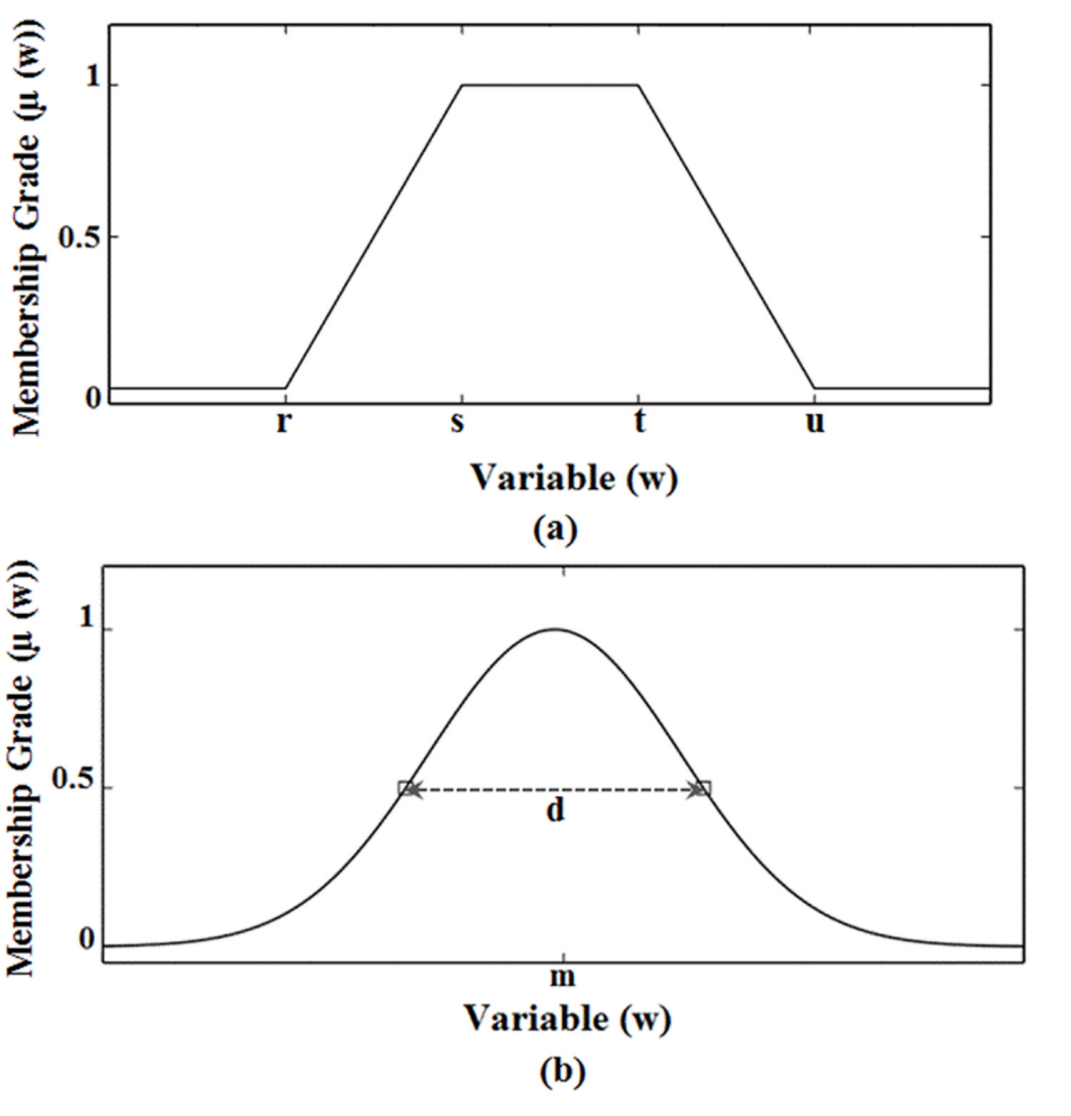
MF plots (a) Trapezoidal, (b) Gaussian.

Where ‘*r*’ ‘s’‘*t*’,’ and *u*’, are the various parameters of trapezoidal MF, and its details are depicted in Fig 4(A).

While the Gaussian MF [30] is expressed as

$$\text{GF}\;\left(\text{w};\text{m},\text{d}\right)={\text{e}}^{-\frac{{\left(\text{w}-\text{m}\right)}^{2}}{2{\text{d}}^{2}}}.$$ (2)

Where ‘*m*’ and ‘*d*’ are the different parameters of the Gaussian MF and its details are shown in Fig 4(B).

### Fuzzy Sets

**Fig 5:**
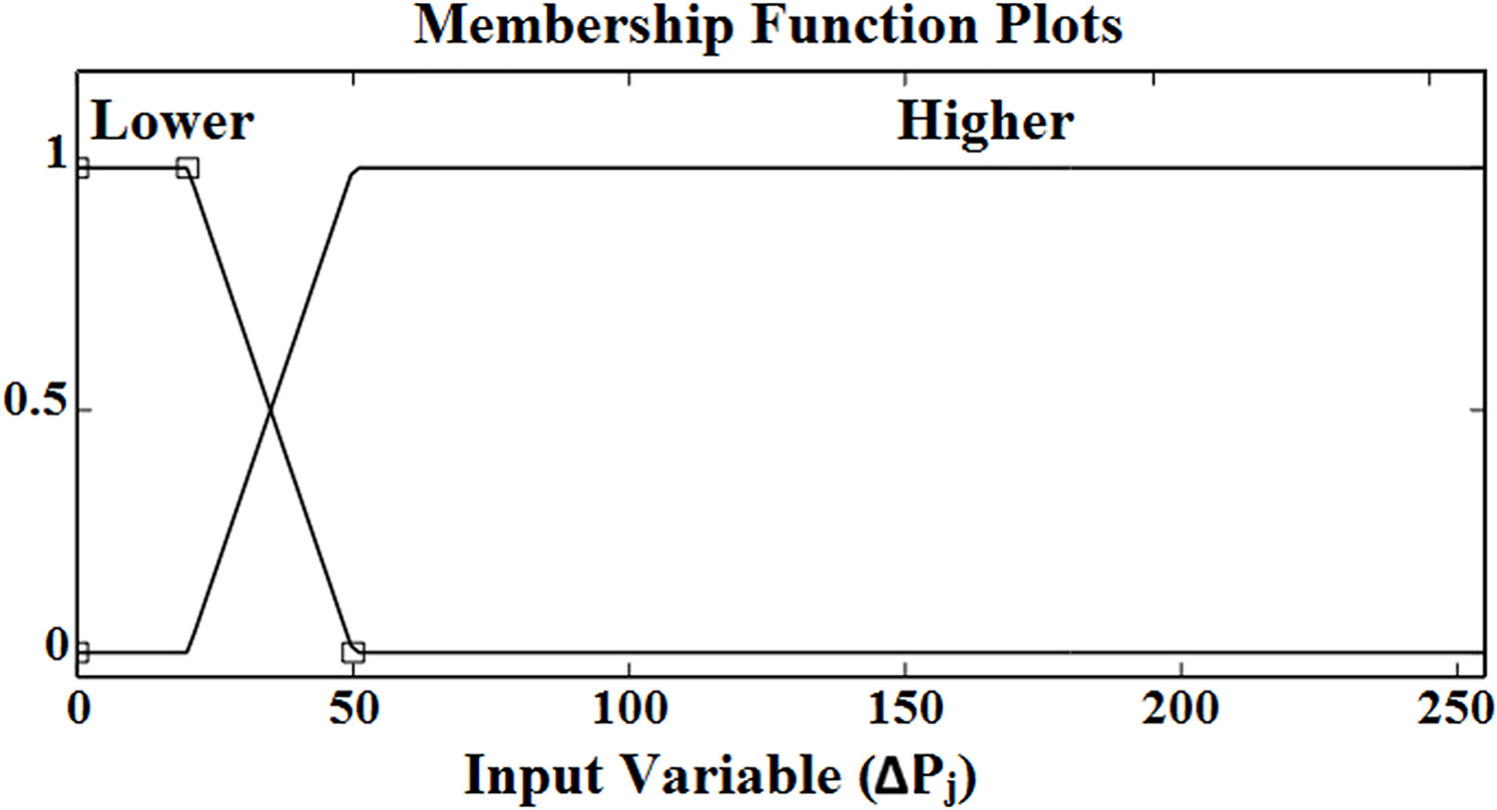
MFs of the input variable *∆P*_*j*_.

**Fig 6:**
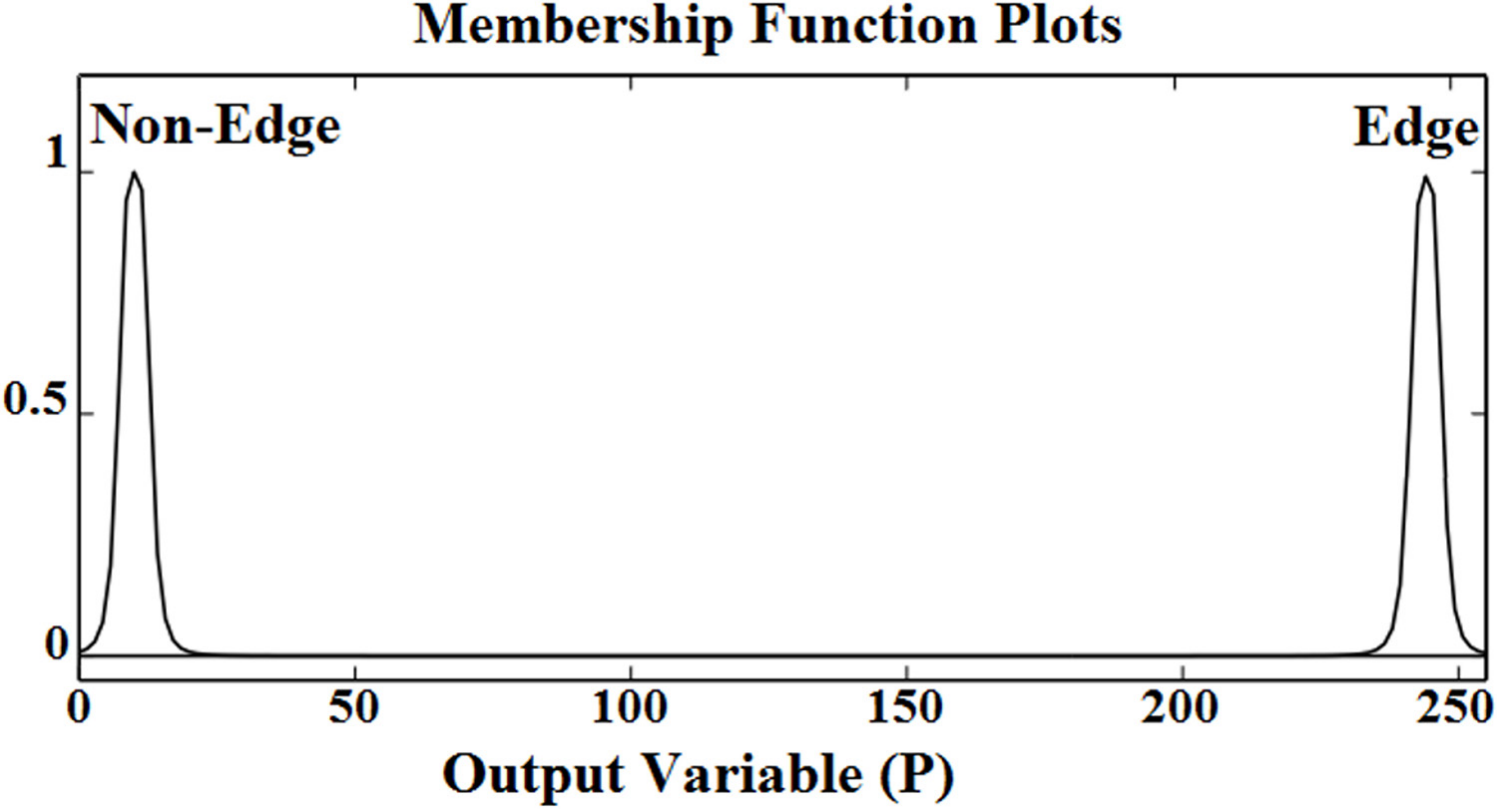
MFs of the output pixel *P*.

Each input, ‘Δ*P*_*j*_’ to fuzzy inference system was divided into two fuzzy sets; *lower* and *higher*. The output (pixel), ‘*P*’ from the fuzzy inference system was divided into two fuzzy sets; *non-edge* and *edge*. The associated MFs with the input and output fuzzy set are shown in s Figs 5 and 6, respectively.

Table 1 lists the various terminologies and parameters of both the input and output fuzzy sets.

**Table 1:** Parameters and terminologies of input and output fuzzy sets.

Linguistic Variable	Parameter	Range	MF Type
***Fuzzy Input ∆P***_***1***_			
Lower	[0 0 25 75]	[0 255]	TMF ^a^
Higher	[25 75 255 255]	[0 255]	TMF ^a^
***Fuzzy Input ∆P***_***2***_			
Lower	[0 0 25 75]	[0 255]	TMF ^a^
Higher	[25 75 255 255]	[0 255]	TMF ^a^
***Fuzzy Input ∆P***_***3***_			
Lower	[0 0 25 75]	[0 255]	TMF ^a^
Higher	[25 75 255 255]	[0 255]	TMF ^a^
***Fuzzy Input ∆P***_***4***_			
Lower	[0 0 25 75]	[0 255]	TMF ^a^
Higher	[25 75 255 255]	[0 255]	TMF ^a^
***Fuzzy Input ∆P***_***5***_			
Lower	[0 0 25 75]	[0 255]	TMF ^a^
Higher	[25 75 255 255]	[0 255]	TMF ^a^
***Fuzzy Input ∆P***_***6***_			
Lower	[0 0 25 75]	[0 255]	TMF ^a^
Higher	[25 75 255 255]	[0 255]	TMF ^a^
***Fuzzy Input ∆P***_***7***_			
Lower	[0 0 25 75]	[0 255]	TMF ^a^
Higher	[25 75 255 255]	[0 255]	TMF ^a^
***Fuzzy Input ∆P***_***8***_			
Lower	[0 0 25 75]	[0 255]	TMF ^a^
Higher	[25 75 255 255]	[0 255]	TMF ^a^
***Fuzzy Output P***			
Non-Edge	[3.5 10]	[0 255]	TMF ^a^
Edge	[3.5 245]	[0 255]	TMF ^a^

^a^ Trapezoidal MF

### Fuzzy Knowledge Base

Fuzzy knowledge base or rule base in fuzzy inference system is a set of linguistic descriptions [31]. Fuzzy rule base plays a key role in fuzzy inference system as it makes conclusions related to either classifying an input or stabilizing and adjusting the output. Fuzzy rule base for the proposed edge detection algorithm consists of the following linguistic descriptions as listed in Table 2.

**Table 2:** Fuzzy knowledge base for the developed edge detection technique.

Rules	Input Variables								Output Variable
	*∆P*_*1*_	*∆P*_*2*_	*∆P*_*3*_	*∆P*_*4*_	*∆P*_*5*_	*∆P*_*6*_	*∆P*_*7*_	*∆P*_*8*_	*P*
1	Higher	Higher	None	None	None	None	None	Lower	Edge
2	Higher	None	None	High	None	None	None	Lower	Edge
3	None	Higher	Higher	None	None	None	None	Lower	Edge
4	None	None	None	Higher	None	Higher	None	Lower	Edge
5	Higher	Higher	None	None	None	None	Lower	None	Edge
6	Higher	None	None	Higher	None	None	Lower	None	Edge
7	None	Higher	Higher	None	None	None	Lower	None	Edge
8	None	None	None	Higher	None	Higher	Lower	None	Edge
9	Higher	Higher	None	None	Lower	None	None	None	Edge
10	Higher	None	None	Higher	Lower	None	None	None	Edge
11	None	Higher	Higher	None	Lower	None	None	None	Edge
12	None	None	None	Higher	Lower	Higher	None	None	Edge

### De-fuzzification

De-fuzzification is the final step involved in fuzzy inference system and is a significant as fuzzification of data set. The membership degrees corresponding to input parameters were attained through fuzzy rule sets and membership functions (MFs). This fuzzy information was quantified into numerical data in this step. There are multiple techniques available for de-fuzzification such as middle of maximum (MOM), center of area (COA), weighted fuzzy mean (WFM), random choice of maximum (RCOM), indexed center of gravity (ICOG), and centre of gravity (COG) etc. Our method employs centroid de-fuzzification (COD), since COD is one of the most accurate, effective and efficient in its applications [32]. The calculated output is as following:

$$c=\frac{\sum_{x=1}^{N}q_{x}z_{x}}{\sum_{x=1}^{N}q_{x}}.$$ (3)

Where *N* is the number of quantized RPN conclusions, ‘*z*_*x*_’ is the support value at which the ‘x^th^’ MF touches its extreme value (it is considered as the centre of maximum range in case of trapezoidal MFs), ‘*q*_*x*_’ is the degree of the truth of the ‘x^*th*^’ MF, and centre of gravity conclusions is indicated by ‘*c*’.

### Contrast Adjustment

**Fig 7:**
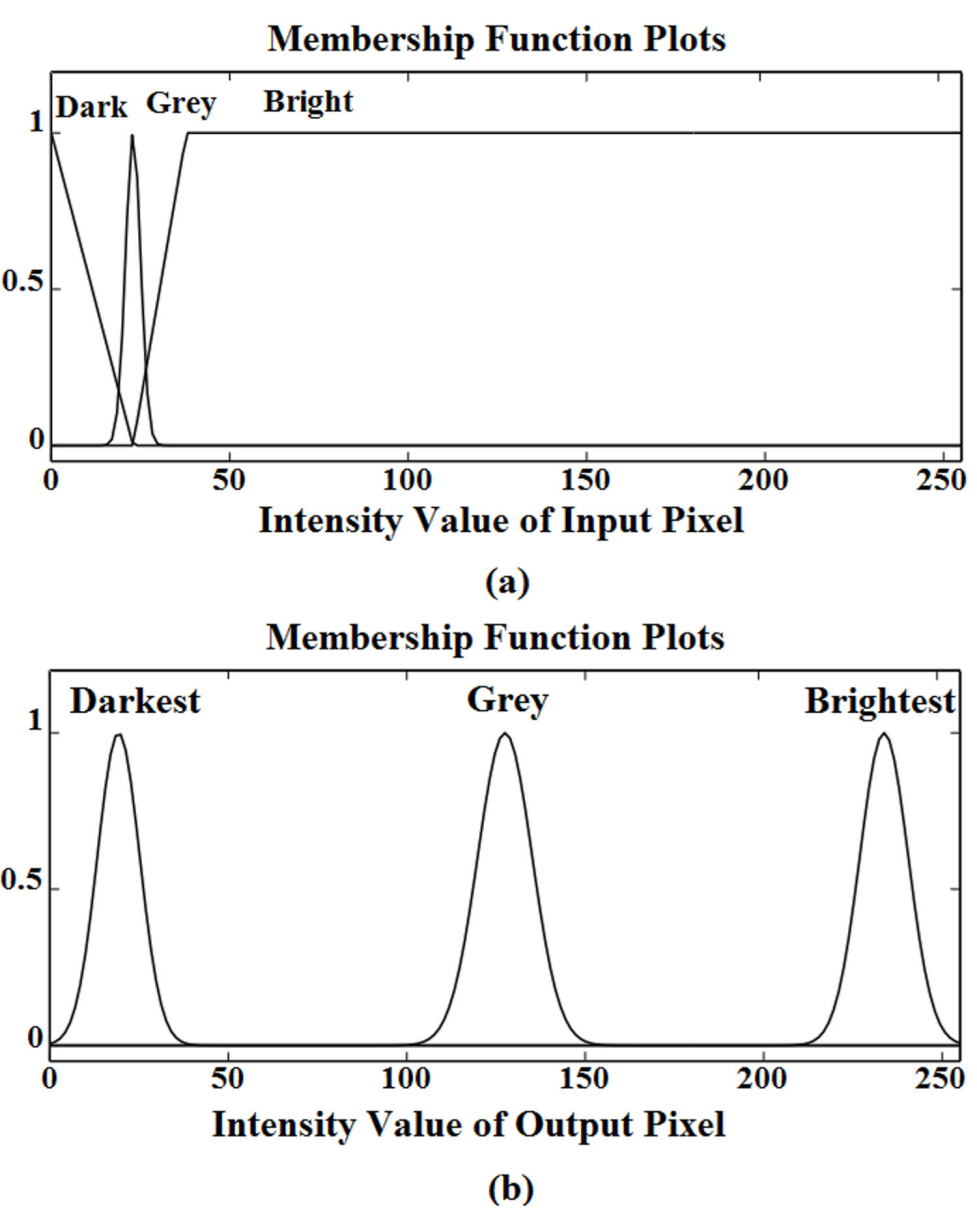
(a) MFs for the intensity value of input pixel (b) MFs for the intensity value of output pixel.

Contrast adjustment was performed before edge detection in the smooth grey (color) clinical images in order to enhance and intensify the edge pixels. The proposed contrast adjustment was based on fuzzy logic. The corresponding MFs for input data (pixels) and output pixels are shown in Fig 7.

The fuzzy rule base for the proposed contrast adjustment is summarized in Table 3.

**Table 3:** Fuzzy rule base for contrast adjustment.

Rules	Input Variable	Output Variable
1	Darker	Darkest
2	Grey	Grey
3	Brighter	Brightest

## Simulation Results and Discussion

The developed edge detection technique was tested on a number of greyscale images including noise free, noisy and smooth images. For noise free and noisy images, only one mask (Edge detection) was employed. However, for smooth clinical images contrast adjustment mask was collectively used, with edge detection mask.

**Fig 8:**
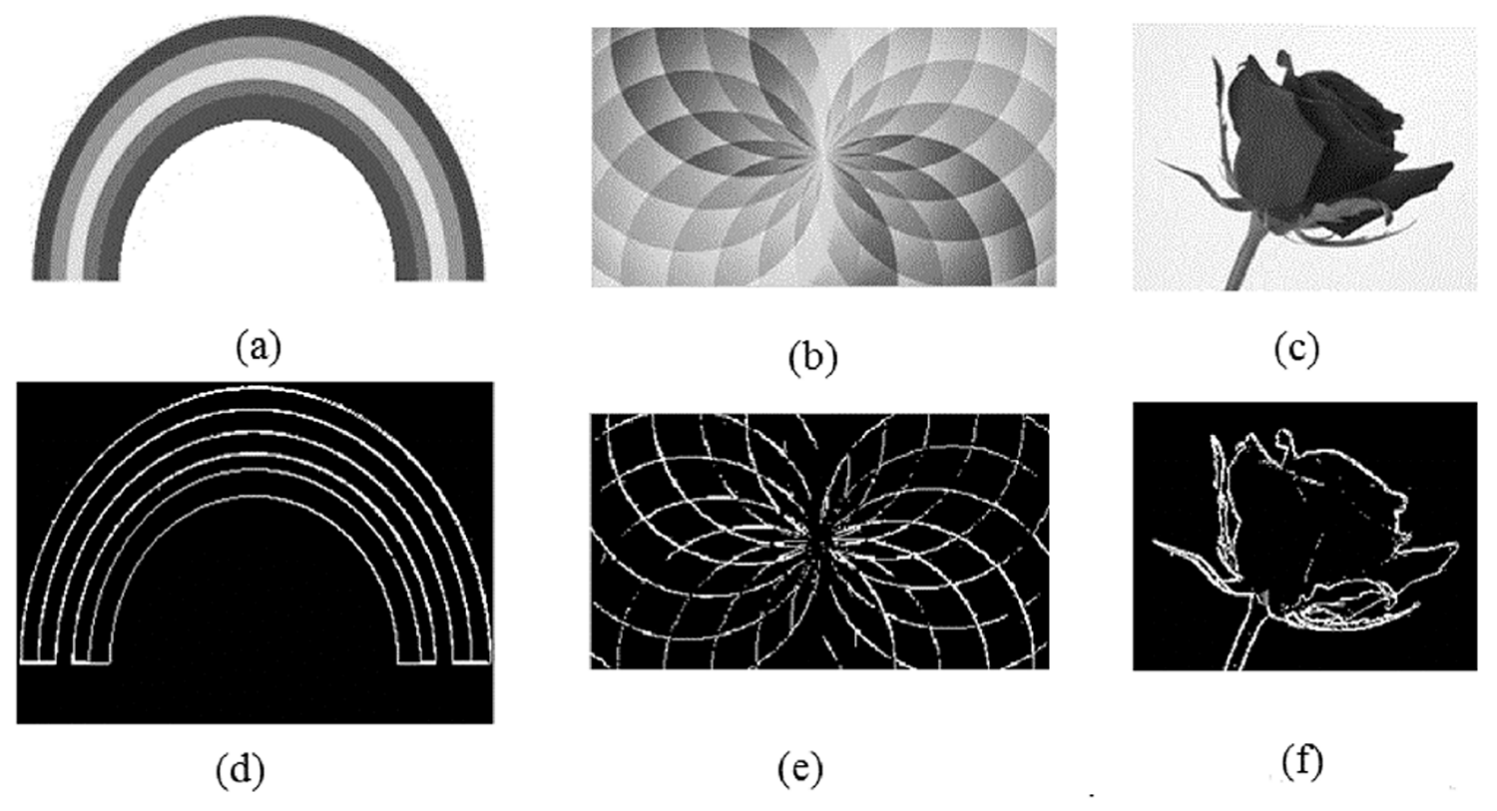
Tested images: (a) Rainbow, (b) Flower, (c) Flower 1, (d) Edge detection in rainbow image, (e) flower image and (f) flower 1 image.

In noise free greyscale images, the developed technique has successfully detected all type of edges as shown in Fig 8. The greyscale rainbow image of size *314*x*192* pixels having five different regions covered by six boundary lines is shown in Fig 8(A). The proposed technique for edge detection have detected these six boundary lines (edges) successfully as shown in Fig 8(D). Similarly, the proposed method has successfully detected edges in the greyscale (flower) images as shown in Fig 8E & 8F.

The developed edge detection technique has the advantage of detecting edges in the noisy images as previously discussed (in the introduction). This was verified by detecting edges in an image having 25 dB ‘salt and pepper’ noise. To compute the noise level in an image through peak signal to noise ratio (PSNR) [33–34], the mean square error (MSE) was first computed as:

$$MSE=\frac{1}{mn}\sum_{v=1}^{n}\sum_{u=1}^{m}\left[G_{1}\left(u,v\right)-G_{2}\left(u,v\right)\right]$$ (4)

Where’ *G*_1_’ and ‘*G*_2_’ represents the input noise free and noisy images respectively. While '*m*' and '*n*' indicates the total number of rows and columns of the input images respectively. Finally the expression for the computation of noise level becomes as following:

$$PSNR=10\;{\text{log}}_{10}\left[\frac{Q_{p}{}^{2}}{MSE}\right]$$ (5)

Where ‘*Q*_*p*_’ denotes the maximum possible intensity value of the pixel in the input image. The value of ‘*Q*_*p*_’ for eight bit unsigned integer data type image is *255*.

**Fig 9:**
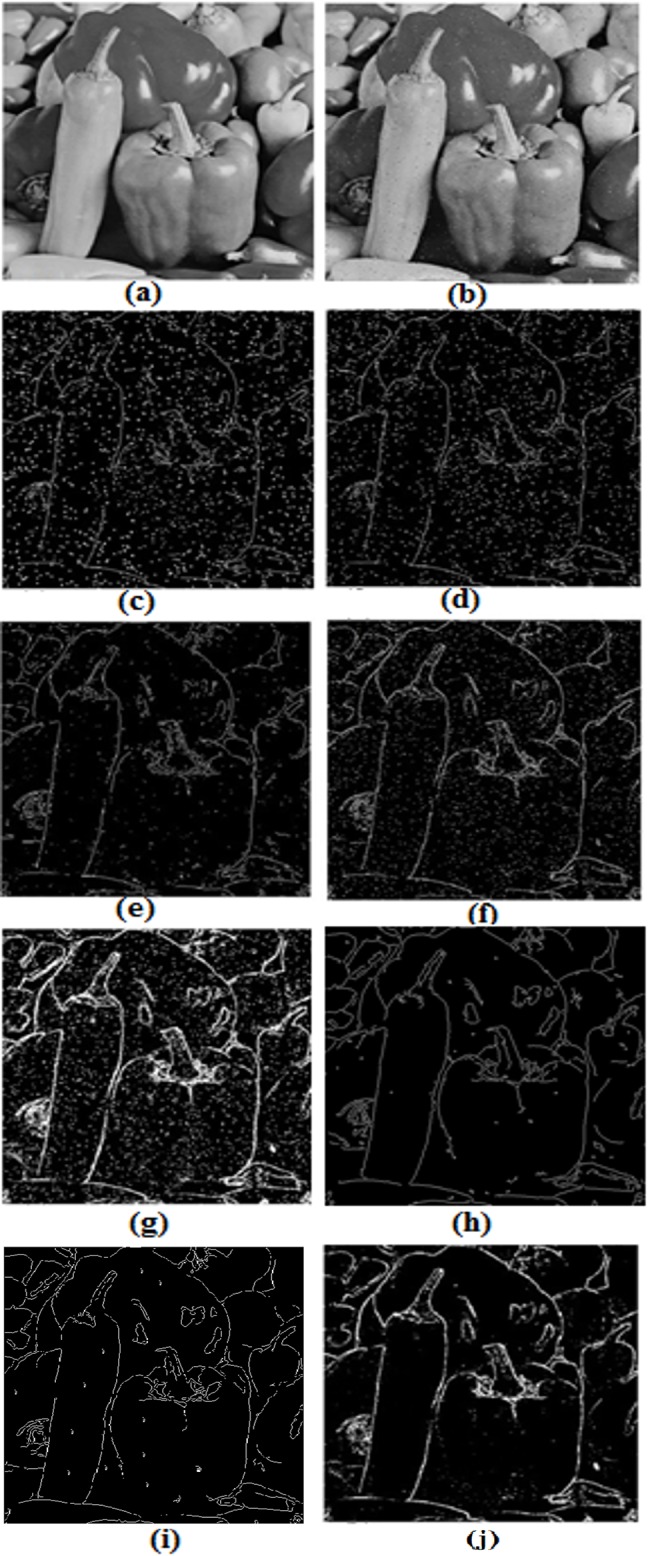
Comparison of experimental results in noisy image: (a) Original image, (b) Noisy image, (c) Sobel edge detection (d) Prewitt edge detection, (e) LoG edge detection, (f) Robert edge detection (g) Previously developed fuzzy based edge detection technique [22], (h) Canny edge detection and (i) The developed method. All the experimentation was performed on image b.

The developed edge detection technique was applied to an image of size *512*x*512* pixels having ‘salt and pepper’ noise at a level of *25*dB. The simulation results are compared with other conventional and reported edge detection algorithms as shown in Fig 9. From the experimental results it is clear that the proposed fuzzy based edge detection algorithm has detected a very few false edge pixels in comparison to the other reported edge detection techniques. The Canny method results were encouraging for this experiment.

**Fig 10:**
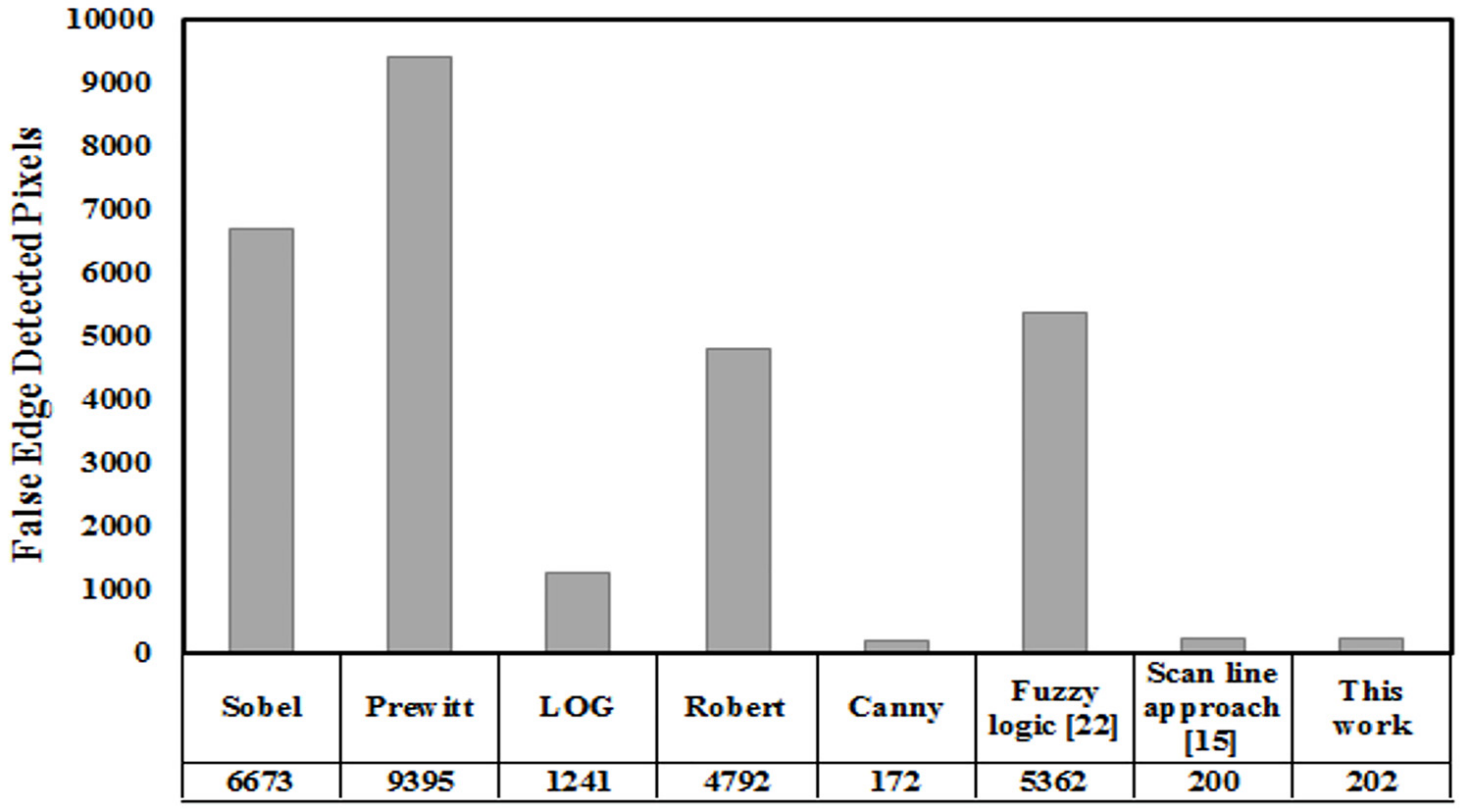
False edge detected pixels in a standard image of *512*x*512* pixels with *25* dB noise level: A comparison.

The number of false edge pixels detected by different reported edge detection techniques is shown in Fig 10. It is evident in Fig 10, that the developed edge detection technique when subject to a noisy image of *512*x*512* size and *25* dB noise level has detected *202* false edge pixels, while other edge detection techniques for instance, Sobel, Prewitt, LOG, Roberts, Canny, previously developed fuzzy logic and scan line approximation [15] based technique, after fine tuning canny method gives few false edge pixels.

**Fig 11:**
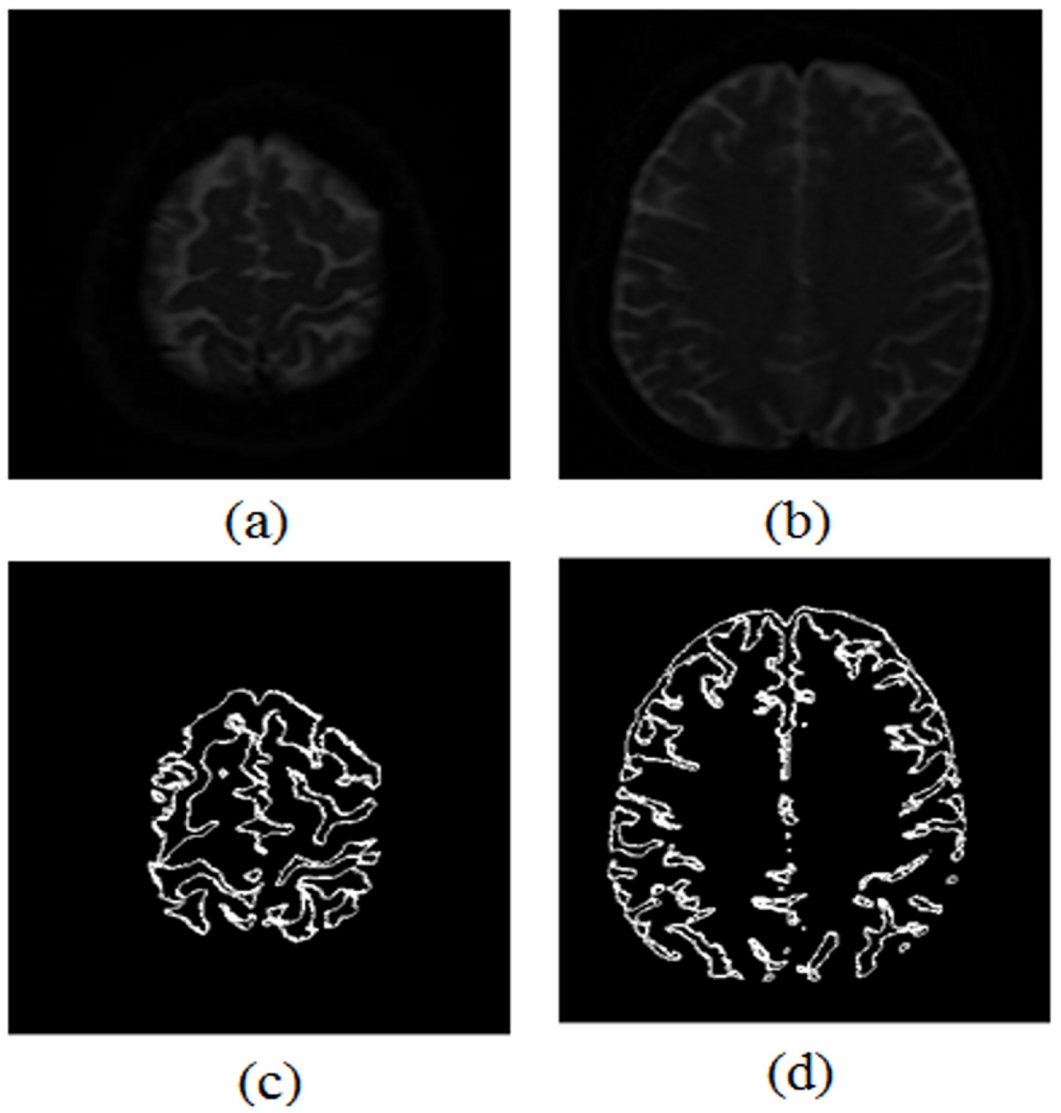
The results of the developed edge detection technique: (a) Greyscale clinical image 1, (b) Greyscale clinical image 2, (c) Edge detection in clinical image1, and (d) Edge detection in clinical image 2.

One of the advantages of the proposed edge detection technique is that it could detect edges in smooth clinical images as well. Fig 11 shows the experimental results of the propose algorithm when applied to smooth clinical MRI images. It is evident in Fig 11 that the developed algorithm has successfully detected edges in the smooth clinical images.

**Fig 12:**
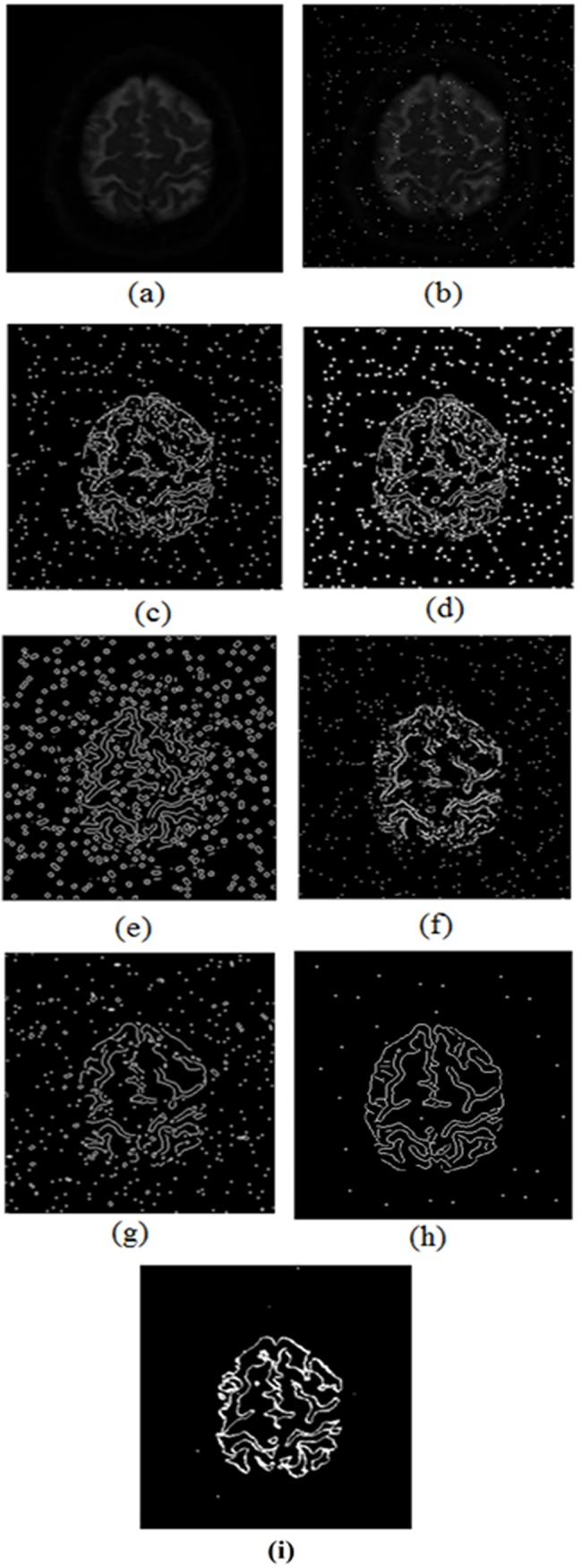
Comparison of the experimental results in noisy clinical image, (a) Original image, (b) Noisy image, (c) Sobel edge detection (d) Prewitt edge detection, (e) LoG edge detection, (f) Robert edge detection (g) Canny edge detection and (h) The developed method.

Finally the developed edge detection technique was applied to the smooth clinical image of size *270*x*290* pixels having *24* dB ‘salt and pepper’ noise. The experimental results were compared with other conventional edge detection techniques like Sobel, Prewitt, LOG, Roberts, Canny and scan line approximation based technique [15] as shown in Fig 12.

**Fig 13:**
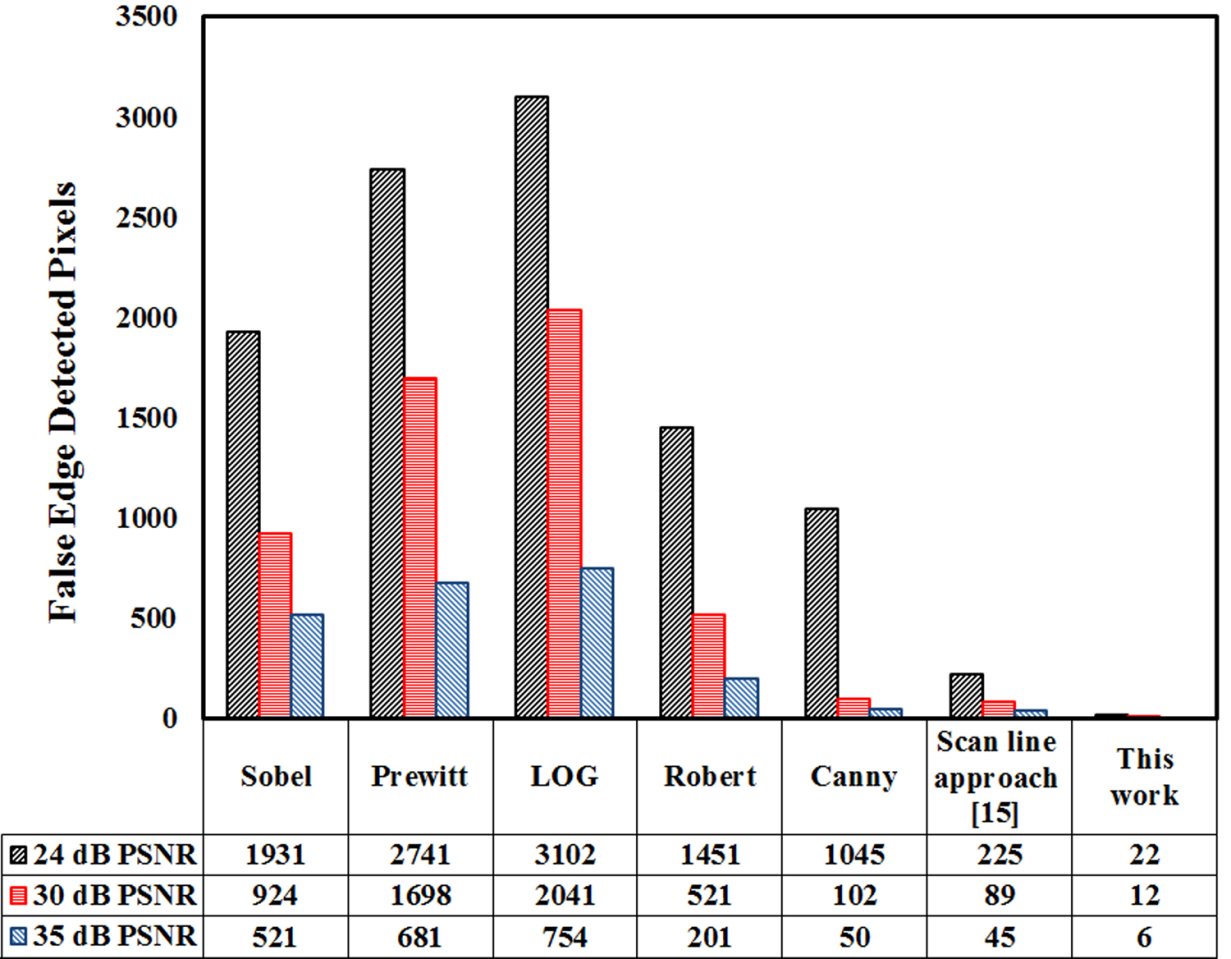
False edge detection in a smooth clinical image of *270*x*290* pixels with *24* dB noise level.

It is clear from results that the developed technique shows excellent results compared to the established edge detection techniques. The number of false edge pixels detected by various edge detection techniques, when subject to images having different PSNR values is shown in Fig 13. It obvious that from Fig 13 that the number of false edge pixels detected by various techniques increases as we increase the noise level in the images. Furthermore, it is clear from Fig 13 that the developed technique when (subjected to image having 24 dB PSNR), has detected very few false edge pixels (*22*) in comparison to the other established edge detection techniques like Sobel (*1931*), Prewitt (*2741*), LOG (*3102*), Roberts (*1451*), Canny (*1045*) and scan line approximation based technique [15] (*225*). Further, Table 4 present statistical analysis such as sensitivity and specificity of the proposed technique with Sobel, Canny and scan line approximation [15]. It is evident from the table that proposed technique has higher value for sensitivity and specificity among the previously established techniques. The proposed technique has potential applications in many disciplines ranging from medical (MRI images, bones defects/cracks) to industrial (surface inspection, crack detection, rust detection) and in agriculture (identification of deforestation, crop yield production, identification of nutritional deficiencies).

**Table 4:** Comparison for sensitivity and specificity

	Sensitivity (%)	Specificity (%)
Sobel	80	89
Canny	81	91
Scan Line [15]	87	94
Proposed Technique	89	96

## Conclusion and Future Work

This paper proposes and demonstrates a fuzzy logic based edge detection algorithm for smooth and noisy images. The developed technique employs a *3*×*3* mask guided by fuzzy rule set for edge detection in noisy images. Furthermore, for smooth clinical images an extra mask of contrast adjustment is integrated with the edge detection mask based on fuzzy logic to intensify the smooth images. The developed technique has successfully detected all the edge pixels in noise free, noisy and smooth images. The developed algorithm is also compared with other conventional and previously developed fuzzy logic based edge detection techniques. The developed edge detection algorithm when subjected to a *512* x *512* size greyscale image having *25* dB ‘salt and pepper’ noise has detected very few false edge pixels (*202*), while the reported edge detection techniques like Sobel, Prewitt, LOG, Roberts, Canny and previously developed fuzzy logic have detected *6673*, *9395*, *1241*, *4792*, *172* and *5362* respectively. When the developed technique was applied to a smooth clinical image of *270* x *290* size having *24* dB ‘salt and pepper’ noise, it detected *22* false edge pixels, while the reported edge detection techniques like Sobel, Prewitt, LOG, Roberts and Canny have respectively detected *1931*, *2741*, *3102*, *1451* and *1045* false edge pixels.It is obvious from the experimental results that in case of smooth and noisy images the developed technique provides better results.

In future work, an investigation on how to incorporate Artificial Immune System and Genetic algorithm with fuzzy logic to develop a hybrid technique for edge detection is under consideration.
